# Genetic kidney diseases – from discovery to precision care

**DOI:** 10.1515/medgen-2025-2049

**Published:** 2026-02-18

**Authors:** Matias Simons, Julia Hoefele

**Affiliations:** Heidelberg University Hospital Institute of Human Genetics Heidelberg Im Neuenheimer Feld 366 69120 Heidelberg Germany; Ludwig-Maximilians University Institute of Human Genetics Goethestr. 29 80336 Munich Germany

In industrialized countries, approximately 10–15 % of the adult population develops chronic kidney disease (CKD). This condition represents a major global health challenge, being associated with substantially increased morbidity and mortality, particularly from cardiovascular complications. In addition to its clinical consequences, CKD imposes considerable economic and logistical burdens on healthcare systems. Once irreversible kidney failure occurs, dialysis or transplantation remain the only therapeutic options, underscoring the need for earlier and more precise diagnosis to slow disease progression and preserve renal function.

Among the diverse etiologies of CKD, genetic kidney diseases constitute a particularly heterogeneous and dynamic field. More than 500 monogenic or otherwise genetically determined disorders have now been described, spanning a broad clinical and molecular spectrum. These include congenital anomalies of the kidney and urinary tract (CAKUT), cystic and ciliopathic diseases, tubular transport disorders, metabolic and stone-forming diseases, complement-mediated nephropathies, and proteinuric glomerular diseases. Recently delineated entities such as autosomal dominant tubulointerstitial kidney disease (ADTKD) have further expanded this spectrum. Many hereditary disorders may also present as part of syndromic or multisystemic conditions with variable renal involvement, where the kidney phenotype represents only one aspect of a broader genetic disease. Despite tremendous progress, many rare nephrogenetic conditions remain incompletely understood at the molecular level.

Clinical presentation is often subtle and unspecific. Early signs such as microscopic hematuria, low-grade proteinuria, or mild renal impairment may precede overt manifestations by years. Morphological abnormalities, including renal cysts or structural malformations, are frequently detected incidentally. In some individuals, hereditary nephropathies remain clinically silent until advanced CKD develops. Nevertheless, an early and accurate genetic diagnosis is crucial. It enables disease-specific management, allows surveillance for extrarenal features, and facilitates targeted genetic counseling and cascade testing within families.

The introduction of next-generation sequencing (NGS), whole-exome (WES), and whole-genome sequencing (WGS) has profoundly changed nephrological diagnostics. Recent studies indicate that up to 25 % of adults with CKD of previously unknown etiology harbor a monogenic cause. Historically, such cases were often classified as hypertensive nephrosclerosis, chronic glomerulonephritis, or interstitial nephritis–reflecting the limitations of purely clinical and histopathologic diagnosis. While genetic testing is already well established in pediatric nephrology, its routine use in adult nephrology is still emerging. Yet, the benefits are evident: a molecular diagnosis enables precise disease classification, informs prognosis, and supports individualized patient management. It also plays a crucial role in transplantation planning and living-donor evaluation, especially in dominantly inherited disorders where clinically unaffected relatives may carry disease-causing variants.

The clinical and therapeutic relevance of molecular diagnosis is increasingly clear. For conditions such as atypical hemolytic uremic syndrome (aHUS), C3 glomerulopathy, and primary hyperoxaluria type 1, unraveling the underlying genetic mechanisms has led to disease-modifying therapies, including complement inhibition and RNA interference–based approaches. Similarly, advances in understanding Alport syndrome, cystinosis, and autosomal dominant polycystic kidney disease (ADPKD) are paving the way for novel, targeted interventions. These developments exemplify how nephrogenetics has evolved from a diagnostic tool into a cornerstone of precision nephrology.

Despite these advances, challenges remain. Variant interpretation, incomplete penetrance, and complex genotype–phenotype correlations continue to complicate clinical translation. The integration of genetic testing into nephrological practice requires close collaboration among (pediatric) nephrologists, geneticists, pathologists, and bioinformaticians. Ethical considerations–such as the management of incidental findings, informed consent, and data protection–further highlight the need for structured genetic counseling. Moreover, equitable access to molecular diagnostics remains a critical issue across healthcare systems.

This issue of *medizinische genetik* brings together five comprehensive contributions that collectively reflect the breadth and depth of the field of nephrogenetics. **Hilger, Westland and Hoefele** provide an in-depth review of the genetic causes of **congenital anomalies of the kidney and urinary tract (CAKUT)**, summarizing recent gene discoveries, developmental pathways, and the clinical implications of genotype–phenotype correlations. **Wenzel, Müller, Erger and Beck** discuss **autosomal dominant tubulointerstitial kidney disease (ADTKD)** as an emerging and conceptually distinct entity, highlighting its genetic heterogeneity, molecular mechanisms, and diagnostic challenges. **Simons and Halbritter** focus on **chronic kidney disease of unexplained cause (CKDx)**, emphasizing the diagnostic yield and clinical impact of systematic genetic testing in adults with previously unclassified CKD. **Milosavljević, Lang and Hermle** presents a detailed overview of **genetic podocytopathies**, tracing the molecular pathways from podocyte injury to glomerulosclerosis and discussing how genetic insights increasingly inform therapeutic decision-making. Finally, **Schumann and Schultheiss** review **the kidney in genetic metabolic disorders**, delineating mechanisms of renal involvement and highlighting how nephrological manifestations can serve as early diagnostic clues in systemic metabolic diseases.

Together, these articles illustrate how the integration of molecular genetics, clinical nephrology, and functional biology continues to refine our understanding of renal pathophysiology. They exemplify the translational continuum from gene discovery to clinical application and underscore the growing importance of genetic diagnostics in everyday nephrological practice. As this field advances, the integration of genomic information into patient care will become increasingly routine. Genetic data will not only improve diagnostic precision but also guide individualized treatment, refine risk prediction, and inform family counseling. Nephrology is entering an era in which genetic knowledge is inseparable from clinical reasoning–a paradigm that promises to transform care for patients and families affected by hereditary kidney diseases.

